# Participation of Integrin ***α***5***β***1 in the Fibronectin-Mediated Adherence of Enteroaggregative *Escherichia coli* to Intestinal Cells

**DOI:** 10.1155/2014/781246

**Published:** 2014-08-07

**Authors:** Mariana Izquierdo, Alejandra Alvestegui, James P. Nataro, Fernando Ruiz-Perez, Mauricio J. Farfan

**Affiliations:** ^1^Centro de Estudios Moleculares, Departamento de Pediatría, Hospital Dr. Luis Calvo Mackenna, Facultad de Medicina, Universidad de Chile, Antonio Varas 360, Providencia, 7500539 Santiago, Chile; ^2^Department of Pediatrics, University of Virginia School of Medicine, Charlottesville, VA 22908, USA

## Abstract

Adherence to the intestinal epithelia is a key feature in enteroaggregative* Escherichia coli *(EAEC) infection. The aggregative adherence fimbriae (AAFs) are involved in EAEC interaction with receptors at the surface of intestinal cells. We and others have demonstrated that fibronectin is a receptor for AAF/II fimbriae. Considering that the major cellular receptor of fibronectin is integrin *α*5*β*1, in this study we evaluated the participation of this receptor in the fibronectin-mediated adherence of EAEC strain 042 to intestinal cells. We found that EAEC strain 042 has the ability to bind directly and indirectly to integrin *α*5*β*1; direct binding was not mediated by AAF/II fimbriae and indirect binding was mediated by AAF/II and fibronectin. Coimmunoprecipitation assays confirmed the formation of the complex AafA/fibronectin/integrin *α*5*β*1. To evaluate EAEC adherence to intestinal cells, T84 cells were incubated with fibronectin and an antibody that blocks the interaction region of integrin *α*5*β*1 to fibronectin, the RGD site. Under these conditions, we found the number of adherent bacteria to epithelial cells significantly reduced. Additionally, fibronectin-mediated adherence of EAEC strain 042 was abolished in HEp-2 cells transfected with integrin *α*5 shRNA. Altogether, our data support the involvement of integrin *α*5*β*1 in the fibronectin-mediated EAEC binding to intestinal cells.

## 1. Introduction

Enteroaggregative* Escherichia coli* (EAEC) is an important cause of endemic and epidemic diarrheal disease worldwide. Adherence to the intestinal epithelia is a key feature of EAEC pathogenesis since it allows bacterial colonization of the intestinal mucosa, the secretion of enterotoxins and cytotoxins, and the induction of proinflammatory cytokines from infected epithelial cells. EAEC adherence to intestinal cells is mediated by fimbrial adhesins, designated aggregative adherence fimbriae (AAFs). Adherence of the prototype EAEC strain 042 to cells and abiotic surfaces requires the AAF pilus variant called AAF/II [1]. Although the importance of the adherence of EAEC to intestinal cells has been established, the cell receptors involved in AAF fimbriae recognition have not been fully characterized. We and others have previously shown recognition of AAF/II fimbriae by extracellular matrix (ECM) protein fibronectin, which enhanced bacterial adherence to the surface of polarized intestinal monolayers [2, 3].

Fibronectin was the first ECM protein to be shown to act as a receptor for bacterial adherence to eukaryotic cells [4]. This glycoprotein is composed of two nearly identical 220 kDa disulfide-bound protein subunits and comprises several structurally distinct domains which can bind to cellular surfaces, fibrin, collagens, DNA, gelatin, integrins, heparin, and heparan sulphate [5]. To date, over 100 bacterial fibronectin binding proteins have been identified and several studies have shown that adhesion to fibronectin would contribute to the successful colonization of bacteria at the epithelial surface, acting as a “molecular bridge” connecting the bacteria with the host cell surface [6].

Integrins are heterodimeric glycoproteins that are critical for a variety of cell-cell and cell-matrix binding events. Once a particular ligand binds to integrin it triggers a specific signaling pathway depending on the type of integrin that is modulated [7]. Fibronectin is a ligand for at least a dozen members of the *β*1 integrin family, where *α*5*β*1 is the major integrin involved in indirect recognition of bacterial pathogens with the ability to bind to fibronectin [6, 8]. For example,* Staphylococcus aureus *employs fibronectin as a pathogenic mechanism to adhere, modulate the intracellular signaling pathway, and invade host cells [7, 9]. Fibronectin-associated bacteria can be recognized by integrin *α*5*β*1 through the RGD region, and this engagement induces the internalization of bacteria by host cells [10, 11].

Considering the above, in this study we sought to characterize the participation of integrin *α*5*β*1 in the fibronectin-mediated adherence of EAEC strain 042 to intestinal cells. Our results support the participation of fibronectin as a bridging molecule allowing the contact of EAEC strain 042 and integrin *α*5*β*1 at the surface of intestinal cells.

## 2. Materials and Methods

### 2.1. Bacterial Strains

Prototype EAEC strain 042 (O44:H18) was originally isolated from a child with diarrhea in Lima, Peru. The EAEC 042*aafA* strain was constructed using the lambda red linear recombination method [12] as previously described [13]. Prototype enterohemorrhagic* E. coli* (EHEC) strain 86-24 and enterotoxigenic* E. coli* (ETEC) strain H10407 were used to evaluate binding to integrin *α*5*β*1. Bacteria were grown overnight in Luria-Bertani (LB) broth with the addition of kanamycin (50 *μ*g/mL) when appropriate.

### 2.2. Cell Lines

T84 and HEp-2 cells were routinely maintained in Dulbecco's modified Eagle's medium (DMEM) F12 and DMEM high glucose (DMEM/HG) (HyClone), respectively, supplemented with 10% fetal bovine serum (FBS), penicillin (10 U/mL), and streptomycin (10 *μ*g/mL), at 37°C under 5% CO_2_.

### 2.3. Solid-Phase Binding Assay

Microtiter plates (Thermo Labsystems, Franklin, MA) were coated overnight at 4°C with a solution of 2 *μ*g/mL of integrin *α*5*β*1 (R&D system) or bovine serum albumin (BSA; Sigma) in integrin binding (IB) buffer containing Tris 25 mM; CaCl_2_ 2 mM; MgCl_2_ 1 mM; MnCl_2_ 1 mM; and NaCl 150 mM, pH 7,5. Unbound protein was removed by washing three times with phosphate buffered saline (PBS, HyClone) and subsequently blocked with 1% skim milk in PBS for 1 h at 37°C. The blocking buffer was removed, and the plates were washed and incubated with a solution of 1 *μ*g/mL of fibronectin (Sigma) in IB buffer by 1 h at 37°C. Binding of fibronectin to integrin *α*5*β*1 was detected using an anti-fibronectin antibody (Sigma), which was incubated for 1 h at room temperature. Anti-rabbit horseradish peroxidase conjugate (HRP; KPL, Gaithersburg, MD) was added following another PBS wash step. Peroxidase activity associated with each well was detected by the addition of 3, 3′, 5, 5′-tetramethylbenzidine (TMB) substrate solution (KPL). Optical densities were read at 450 nm with a 96-well plate reader (Infinite, Tecan). To determine the adhesion of EAEC or AafA-*dsc* protein to integrin *α*5*β*1 and fibronectin/integrin *α*5*β*1 complex we performed the same protocol described above with some modification. After the incubation with fibronectin, plates were washed three times with PBS and incubated with bacteria in a 200 *μ*L final volume or with a solution of 1 *μ*g/mL of AafA-*dsc* protein. Incubation with bacteria was performed for 2 h at 37°C while incubation with protein was carried out for 2 h at room temperature. Plates were washed and rabbit anti-O44 serum (Denka Seiken Co., LTD, Tokyo, Japan) diluted 1 : 200 or rabbit anti-AafA 1 : 2000, respectively, was added to the wells and incubated for 1 h at room temperature. Anti-rabbit HRP conjugate was added following another PBS wash step and peroxidase activity associated with each well was detected by the addition of TMB substrate solution. Optical densities were read at 450 nm.

### 2.4. Coimmunoprecipitation Assays

We constructed columns with anti-AafA antibodies and control columns (without antibodies) using pierce coimmunoprecipitation (Co-IP) kit following manufacturer instructions. To evaluate the formation of the AafA/fibronectin complex, we prepared a solution of 5 *μ*g/mL of AafA-*dsc* protein and 10 *μ*g/mL of purified fibronectin. This mixture was incubated with gentle shaking for 1 h at room temperature and then added to each column following manufacturer instructions. Unbound protein was removed by washing three times with PBS and immunoprecipitated proteins were eluted. To evaluate the formation of the complex AafA/fibronectin/integrin *α*5*β*1, we performed the same procedure described above. We incubated 5 *μ*g/mL of AafA-*dsc*, 10 *μ*g/mL of purified fibronectin, and 5 *μ*g/mL of purified integrin *α*5*β*1 protein for 1 h at room temperature. Fractions recovered from control column and column with anti-AafA antibodies were analyzed by Dot blot.

### 2.5. Dot Blot

Nitrocellulose membrane was washed with PBS and then coated with 10 *μ*L of fractions recovered from control column and column with anti-AafA antibodies. The membrane was blocked with BSA 1% for 1 h at room temperature and the presence of AafA-*dsc*, fibronectin, or integrin *α*5*β*1 proteins was determined using anti-AafA, anti-fibronectin (Sigma), or anti-integrin *α*5 (Santa Cruz Biotechnology). Membranes were washed three times with PBS-Tween 0,05% and anti-rabbit HRP was added. Peroxidase activity associated was detected by the addition of TMB substrate solution directly on membrane. The resulting dots were scanned and signal intensity was quantified using UN-SCAN-IT 6.1 software.

### 2.6. Adherence Assays

Epithelial cells, grown in 96-well plates, were incubated for 30 min with 100 *μ*L/well of DMEM-F12 only or supplemented with fibronectin (1 *μ*g/well), antibodies anti-integrin *α*5*β*1 that block the interaction with RGD site in fibronectin (Abcam) or anti-integrin *α*5 (Santa Cruz Biotechnology). Then, medium was aspirated and bacteria were added to the monolayer (in triplicate), with a multiplicity of infection (MOI) of 10. The plates were incubated at 37°C in 5% CO_2_ for 3 h and then washed three times with PBS. Cells were lysed with a solution containing 0.1% (v/v) Triton X-100/PBS and serial dilutions of the lysates were plated on LB agar. The number of adherent bacteria was determined by counting colonies forming units (CFU).

### 2.7. shRNA

Subconfluent cultures (~40–50%) of HEp-2 cells grown in 6-well plates were transfected with small hairpin RNA (shRNA) for integrin *α*5 (sc-29372-SH; Santa Cruz Biotechnology) following the manufacturer's instructions (Santa Cruz Biotechnology). A solution of DMEM/HG, without antibiotics or serum, containing shRNA transfection reagent (Santa Cruz Biotechnology) and 1 *μ*g of integrin *α*5 shRNA or scrambled shRNA was added to HEp-2 cells, which were then incubated for 7 h at 37°C in 5% CO_2_. Later, medium was replaced by DMEM supplemented with FBS and antibiotics, and after 48–72 h transfected cells were selected by puromycin resistance. To verify a reduction in integrin *α*5 expression, total RNA of HEp-2 cells transfected was obtained using total RNA I (Omega Biotech) and treated with RNase-free DNase I (Omega Biotech). Two micrograms of RNA was reverse-transcribed by using kit Affinity Script PCR cDNA (Stratagene). The synthesized cDNA was used to quantify the expression of integrin *α*5, using the following primers: forward 5′ TGCAGTGTGAGGCTGTGTACA 3′ and the reverse 5′-GTGGCCACCTGACGCTC-3′. Levels of mRNA expression were normalized to those of the human housekeeping gene glyceraldehyde 3-phosphate dehydrogenase (*GAPDH*). Changes in cycle threshold (ΔCT) values for each gene were obtained by subtracting the mean threshold cycle (CT) of the reference* GAPDH* gene (data not shown).

### 2.8. Statistical Analysis

Statistical significance between the individual groups was analyzed using the unpaired Student's *t*-test with a threshold of *P* < 0.05. Values are expressed as the means ± the standard errors of the mean of three experiments.

## 3. Results

### 3.1. Diarrheagenic* E. coli* Binding to Integrin *α*5*β*1

**Figure 1:**
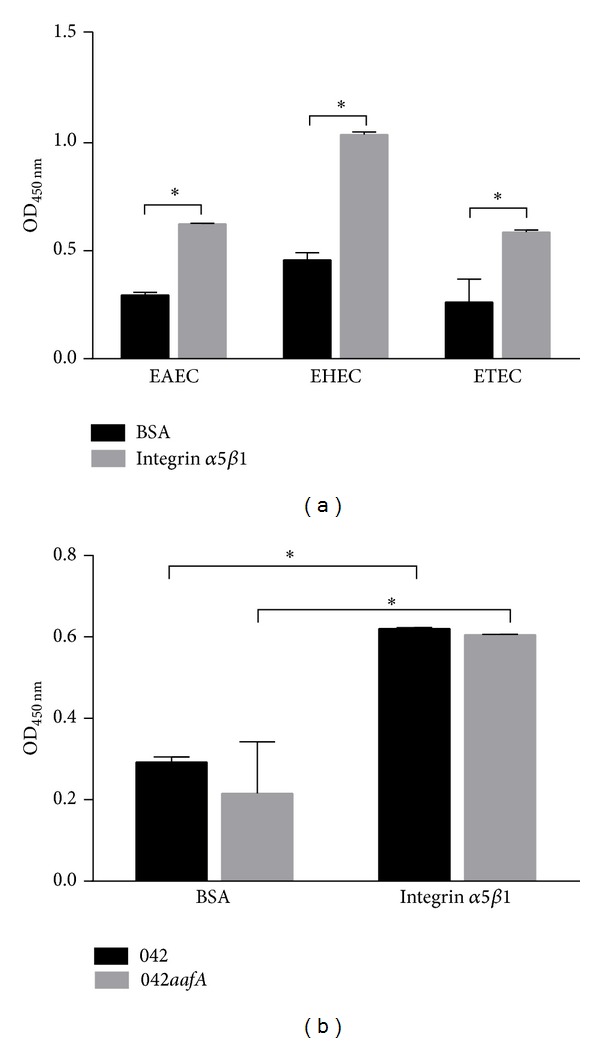
Diarrheagenic* E. coli* bind to integrin *α*5*β*1. (a) EAEC, EHEC, and ETEC were added to integrin *α*5*β*1- and BSA-coated wells and bacterial binding was detected by ELISA, using anti-O44, anti-O157, and anti-O78 serum, respectively. (b) EAEC strain 042 and 042*aafA *were added to integrin *α*5*β*1- and BSA-coated wells. EAEC binding was detected by ELISA, using anti-O44 serum. *Significantly different between treatments (*P* < 0.05).

First, we evaluated binding to integrin *α*5*β*1 of three strains of diarrheagenic* E. coli*: EAEC, EHEC, and ETEC. We obtained that all strains studied bind significantly to purified integrin *α*5*β*1-coated wells compared to BSA-coated wells, used as negative control (Figure 1(a)). To characterize the mechanism involved in the EAEC strain 042 binding to integrin *α*5*β*1, we quantified binding of EAEC strain 042 and 042*aafA* mutant to recombinant purified human integrin *α*5*β*1 by ELISA. Binding of EAEC strain 042 and 042*aafA* to integrin *α*5*β*1-coated wells was significantly higher than to BSA-coated wells, but no difference in the binding to integrin *α*5*β*1 between EAEC strain 042 and 042*aafA* was observed (Figure 1(b)), suggesting that AAF/II fimbriae have no direct role in the binding of EAEC to integrin *α*5*β*1.

### 3.2. Participation of Fibronectin in the Binding of EAEC Strain 042 to Integrin *α*5*β*1

**Figure 2:**
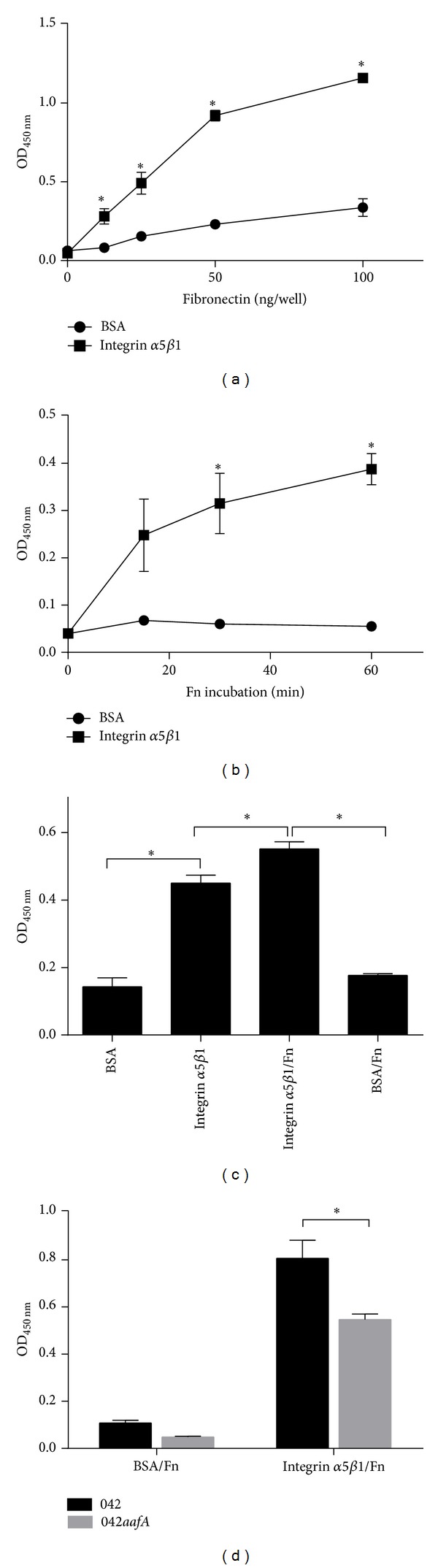
Fibronectin/integrin *α*5*β*1 complex increases the adhesion of EAEC strain 042. (a) Integrin *α*5*β*1- and BSA-coated wells were incubated with increasing concentrations of fibronectin (25, 50, and 100 ng) or (b) with 100 ng of fibronectin (Fn) for 15, 30, or 60 min. Bound fibronectin was detected by ELISA, using anti-fibronectin antibodies. (c) EAEC 042 was added to integrin *α*5*β*1- and BSA-coated wells incubated or not with fibronectin. (d) EAEC strain 042 and 042*aafA* were added to integrin *α*5*β*1- and BSA-coated wells incubated with fibronectin. EAEC binding was detected by ELISA using anti-O44 serum. The bars represent the mean for three experiments, with the error bars indicating standard deviation. *Significantly different between treatments (*P* < 0.05).

First, we assessed the binding of increasing concentrations of fibronectin to integrin *α*5*β*1 and BSA-coated wells used as controls. We found that fibronectin binds significantly to integrin *α*5*β*1-coated wells at all concentrations tested in a dose-dependent manner (Figure 2(a)). Later, we evaluated the time required for binding of fibronectin to integrin *α*5*β*1 and we found that fibronectin binds significantly to integrin *α*5*β*1-coated wells after 30 min of incubation (Figure 2(b)). From these assays, we decided to use 100 ng of fibronectin for 60 min to evaluate the binding of EAEC strain 042 to fibronectin/integrin *α*5*β*1 complex by ELISA. EAEC strain 042 binding to the complex fibronectin/integrin *α*5*β*1 was significantly higher than the binding to integrin *α*5*β*1 alone (Figure 2(c)). Considering that EAEC strain 042 binding to BSA or BSA incubated with fibronectin was similar, we dismissed the possibility that the increase observed in the presence of the complex fibronectin/integrin *α*5*β*1 was due to fibronectin incubation. Later, we evaluated the participation of the AAF/II fimbriae in the binding of EAEC strain 042 to the complex fibronectin/integrin *α*5*β*1, and we found that binding of EAEC 042*aafA* mutant strain was significantly lower compared to the binding of wild-type EAEC strain 042 (Figure 2(d)).

### 3.3. Formation of Complex AafA/Fibronectin/Integrin *α*5*β*1

**Figure 3:**
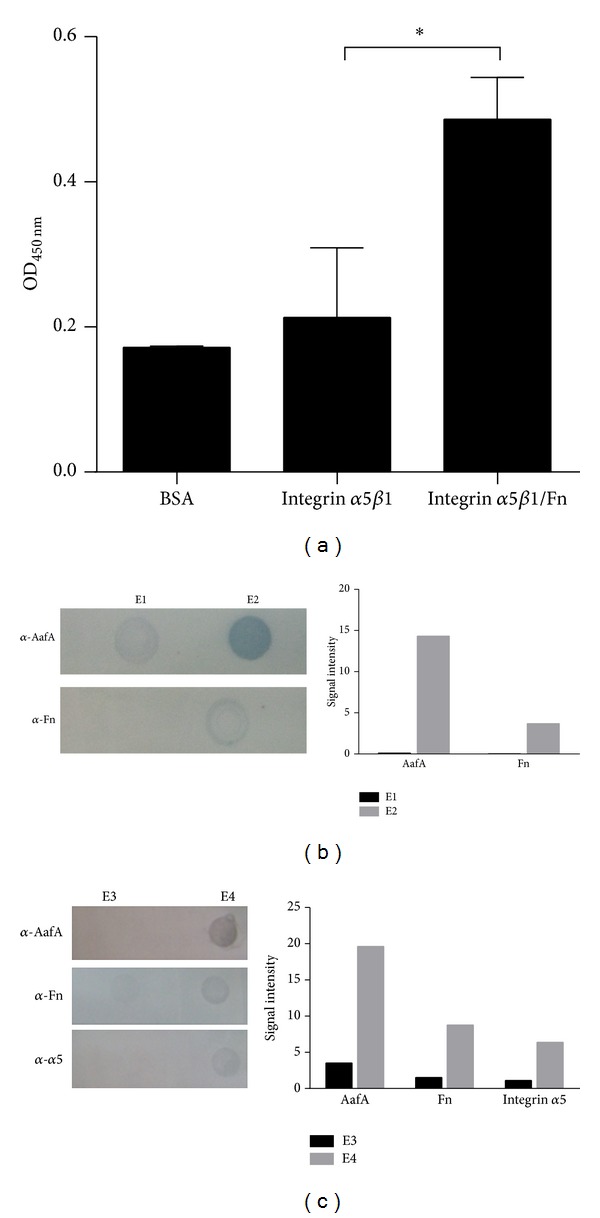
AafA binding to fibronectin/integrin *α*5*β*1 complex. (a) AafA-*dsc* protein was added to integrin *α*5*β*1- and BSA-coated wells incubated or not with fibronectin (Fn). AafA-*dsc* binding was detected by ELISA using anti-AafA antibodies. The bars represent the mean for three experiments, with the error bars indicating standard deviation. *Significantly different between treatments (*P* < 0.05). (b) AafA-*dsc* and fibronectin or (c) AafA-*dsc*, fibronectin, and integrin *α*5*β*1 were mixed and the complex formed was added to control column or a column with anti-AafA antibodies for coimmunoprecipitation analysis. The eluted fraction obtained from control column (E1 and E3) or a column with anti-AafA antibodies (E2 and E4) was analyzed by Dot blot, using anti-AafA, anti-integrin *α*5, and anti-fibronectin antibodies. The resulting autoradiography was scanned and signal intensity was quantified by UN-SCAN-IT 6.1 software. One representative experiment of three independent experiments with similar result is shown.

EAEC strain 042 binding to fibronectin is mediated by the major subunit of the AAF/II fimbriae, the AafA protein [2]. We evaluated the binding of purified AafA-*dsc* protein to integrin *α*5*β*1 and fibronectin/integrin *α*5*β*1 complex. No direct binding of AafA-*dsc* protein to integrin *α*5*β*1 was found; however, a significant binding to fibronectin/integrin *α*5*β*1 complex was observed (Figure 3(a)). These observations were confirmed by coimmunoprecipitation assays of a mixture containing the purified AafA-*dsc* and fibronectin. Dot blot analysis showed the presence of AafA and fibronectin in the fraction obtained from a column with anti-AafA antibodies and not in the control column (Figure 3(b)). To evaluate the formation of the complex AafA-*dsc*/fibronectin/integrin *α*5*β*1, these proteins were incubated and immunoprecipitated using anti-AafA antibodies. In the immunoprecipitated fraction with anti-AafA antibodies the three proteins were found in higher amount compared to the proteins present in the fraction of control column (Figure 3(c)).

### 3.4. Role of Integrin *α*5*β*1 in the Adherence of EAEC Strain 042 to Cells

**Figure 4:**
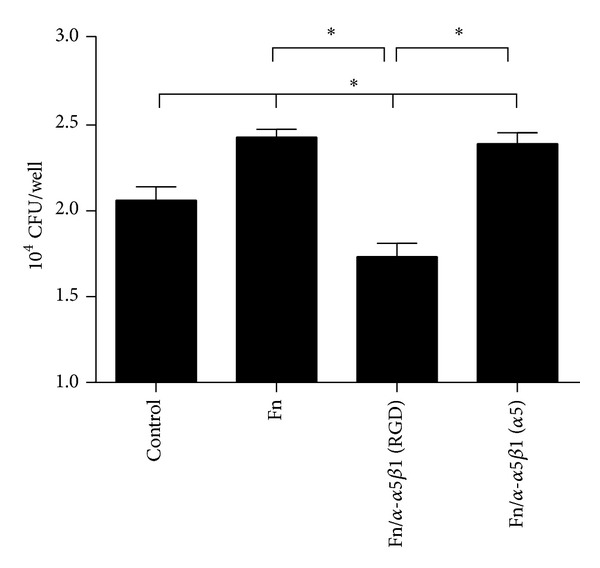
Adhesion of EAEC strain 042 to T84 cells. T84 cells preincubated with DMEM only (control) or medium supplemented with fibronectin (Fn), Fn and anti-integrin *α*5*β*1 (RGD), or anti-integrin *α*5*β*1 (*α*5) were infected with EAEC strain 042. The number of adherent bacteria was determined by colony forming unit counts (CFU). *Significantly different between treatments (*P* < 0.05).

To determine the participation of integrin *α*5*β*1 in the fibronectin-mediated adherence of EAEC strain 042 to intestinal epithelial cells, prior to infection, we incubated T84 cells with fibronectin in the presence of two different antibodies: a polyclonal antibody against several epitopes of the integrin *α*5 molecule and a monoclonal antibody that only blocks the fibronectin-integrin *α*5*β*1 interaction site, the RGD site. EAEC strain 042 adherence to cells preincubated with fibronectin was significantly higher than cells incubated with medium only used as control, confirming previous observations [2]. EAEC strain 042 adherence was significantly lower when cells were incubated with fibronectin and anti-integrin *α*5*β*1 (RGD) antibodies, compared to cells incubated with fibronectin alone. The incubation with fibronectin and anti-integrin *α*5 antibodies did not affect the adherence to cells compared to cells incubated only with fibronectin (Figure 4).

**Figure 5:**
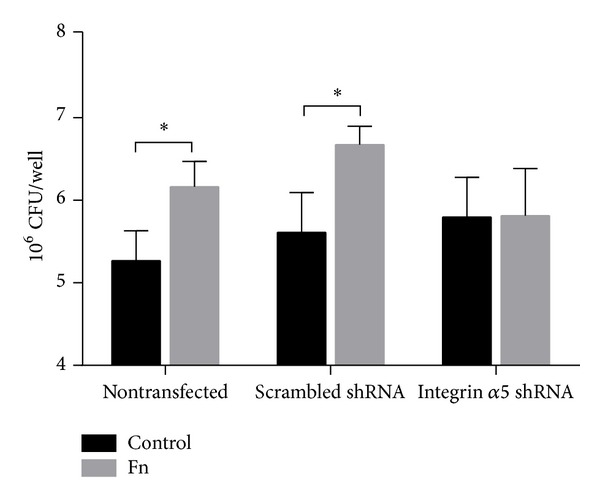
Integrin *α*5 expression knockdown reduces EAEC strain 042 fibronectin-mediated binding to epithelial cells. HEp-2 cells nontransfected and transfected with scrambled or integrin *α*5 shRNA were preincubated with DMEM only (control) or medium supplemented with fibronectin (Fn) and then infected with EAEC strain 042. Numbers of adherent bacteria were determined by colony forming unit counts (CFU). *Significantly different compared to control treatment (*P* < 0.05).

To confirm the participation of integrin *α*5*β*1 in the adherence of EAEC strain 042 to epithelial cells, HEp-2 cells were transfected with scrambled shRNA or integrin *α*5 shRNA. Prior to EAEC infection, cells were preincubated with DMEM or medium supplemented with fibronectin. The significant increase in EAEC adherence to nontransfected HEp-2 cells or HEp-2 cells transfected with scrambled shRNA in presence of fibronectin compared to cell not incubated with this ECM protein was not observed in HEp-2 cells transfected with integrin *α*5 shRNA (Figure 5).

Altogether, these results support the role of integrin *α*5*β*1 in the fibronectin-mediated EAEC adherence to intestinal cells.

## 4. Discussion

Adherence to intestinal epithelia is a key process in EAEC pathogenesis. Although the cellular receptors involved in the EAEC recognition are not fully characterized, previous studies have demonstrated that fibronectin is a receptor for the AAF/II fimbriae [2, 3], one of the major adherence factors of EAEC. In the prototype EAEC strain 042 carrying the AAF/II fimbriae variant, this binding is mediated by AafA protein. Fibronectin can bind different receptors in the eukaryotic cell, being integrin *α*5*β*1 the most studied. This interaction has different roles* in vivo,* such as fibronectin fibril formation, extracellular matrix assembly, and transduction of biochemical signals that regulate cell adhesion, migration, proliferation, and apoptosis [14].

Fibronectin-integrin *α*5*β*1 interaction occurs through a short polypeptide sequence Arg-Gly-Asp or RGD sequence present in the fibronectin molecule which interacts with the interface between subunits *α* and *β* of integrin *α*5*β*1 [15]. In this study, we evaluated the interaction between fibronectin and integrin *α*5*β*1 using purified proteins and we found that this interaction is dependent on the fibronectin concentration employed and the period of incubation. Next, we evaluated the participation of integrin *α*5*β*1 in the adherence of EAEC, and we found that prototype EAEC strain 042 binds to purified integrin *α*5*β*1 but this binding is independent of the AAF/II fimbriae, suggesting the contribution of other adhesins in this interaction (Figure 1). The participation of integrins as pathogen receptors has been described previously. For example, nonfimbrial adhesins AFA (homologous to AAF fimbriae) present in pathogenic* E. coli* strains have the ability to bind to *β*1 integrin [16]. In uropathogenic* E. coli* (UPEC) strains, type 1 fimbriae bind to integrins *β*1 and *α*3 and the heterodimer formed between these proteins [17]. In Gram-positive pathogens, binding to integrin *α*5*β*1 is mediated by fibronectin. This protein acts as a molecular bridge connecting bacteria with host cell. Fibronectin binding proteins (FnBP) located at the surface of bacteria bind to fibronectin, and the complex bacteria/fibronectin is recognized by integrin *α*5*β*1 promoting the endocytosis of bacteria [10]. Our data showed an increase in the binding of EAEC strain 042 to the complex fibronectin-integrin *α*5*β*1 (Figure 2), suggesting that EAEC has the ability to bind to integrin *α*5*β*1 either directly or mediated by fibronectin. Similarly, we found that AafA-*dsc* protein binds to fibronectin/integrin *α*5*β*1 complex but not to integrin *α*5*β*1 alone (Figure 3). Altogether, these results suggest that EAEC can bind directly (not mediated by AAF/II fimbriae) and indirectly (mediated by AAF/II and fibronectin) to integrin *α*5*β*1. The latter observation is supported by coimmunoprecipitation assays using purified AafA-*dsc* protein that confirmed the formation of the complex AafA/fibronectin/integrin *α*5*β*1 (Figure 3). To evaluate the importance of this interaction in the context of EAEC infection to intestinal cell, we assessed the adherence of EAEC strain 042 to T84 cells by preincubating with an antibody that blocks the RGD binding site of integrin *α*5*β*1 and by knocking down the integrin *α*5 expression with shRNA. Under these conditions, fibronectin-mediated EAEC adherence was abolished (Figures 4 and 5).

In the intestinal epithelia, fibronectin and integrins are principally expressed in the basolateral surface. However, in inflamed intestines upregulation in the expression of these molecules occurs, promoting their presence in the apical surface of cells, thus enabling interaction with bacteria [18–20]. Previous studies have demonstrated that EAEC induces the expression and release of proinflammatory cytokines and loss of the epithelial barrier [21, 22]. In this condition, the ability to bind to the fibronectin/integrin *α*5*β*1 complex might be involved in the aggregative adherence phenotype displayed by EAEC on intestinal cells.

In conclusion, we described a novel potential receptor for EAEC strain 042, integrin *α*5*β*1, which might be an important factor in the fibronectin-mediated adherence of EAEC strain 042 to intestinal cells.
